# Connecting the Lines between Hypogonadism and Atherosclerosis

**DOI:** 10.1155/2012/793953

**Published:** 2012-02-16

**Authors:** Akl C. Fahed, Joanna M. Gholmieh, Sami T. Azar

**Affiliations:** ^1^Department of Genetics, Harvard Medical School, 77 Avenue Louis Pasteur, Boston, MA 02115, USA; ^2^School of Pharmacy, Lebanese American University, P.O. Box 36, Byblos, Lebanon; ^3^Department of Internal Medicine, American University of Beirut, Bliss Street, P.O. Box 11-0236, Beirut, Lebanon

## Abstract

Epidemiological studies show that atherosclerotic cardiovascular disease is a leading cause of morbidity and mortality worldwide and point to gender differences with ageing males being at highest risk. Atherosclerosis is a complex process that has several risk factors and mediators. Hypogonadism is a commonly undiagnosed disease that has been associated with many of the events, and risk factors leading to atherosclerosis. The mechanistic relations between testosterone levels, atherosclerotic events, and risk factors are poorly understood in many instances, but the links are clear. In this paper, we summarize the research journey that explains the link between hypogonadism, each of the atherosclerotic events, and risk factors. We look into the different areas from which lessons could be learned, including epidemiological studies, animal and laboratory experiments, studies on androgen deprivation therapy patients, and studies on testosterone-treated patients. We finish by providing recommendations for the clinician and needs for future research.

## 1. Introduction

Atherosclerotic cardiovascular disease is the leading cause of mortality and morbidity worldwide. With the advances in medicine leading to increasingly ageing populations, cardiovascular disease appears as the number one noncommunicable cause of death worldwide, and it is on the rise [1]. Atherosclerosis is a diffuse disease process with many risk factors. Age, dyslipidemia, hypertension, obesity, and diabetes are some of the risk factors that have been extensively studied. Additionally, males have double the risk of females [2]. This gender difference was initially thought to be exclusively due to female atheroprotective hormones. Nonetheless, a large part of this gender susceptibility difference is due to the decreasing androgen levels in ageing men, who are at highest risk for atherosclerosis [3].

Hypogonadal hypogonadism is a commonly undiagnosed disease in the general population with symptoms such as decreased libido, fatigue, muscle loss and increased visceral fat, osteoporosis, reduced energy, and mood changes. More importantly, hypogonadism is associated with increased atherosclerosis. This relationship is very complex and hence is a subject for tremendous amount of research. Hypogonadism has been associated with all the risk factors of atherosclerosis mentioned above and contributes to the increased susceptibility of men to the disease [4]. The causality relation, however, is still controversial due to the poorly understood mechanisms. Also very often, the relationship between hypogonadism and the atherosclerotic risk factor is bidirectional. More importantly, hypogonadism has been implicated in the pathogenesis of atherosclerosis and the susceptibility of the myocardium to ischemia [5]. Various animal and *in-vitro* studies have elucidated the pathways through which testosterone affects vascular and myocardial health. Although a lot of research progress has been made in that regard, the story is still far from being complete with many statistical associations in men with poorly understood mechanisms. As androgen deficiency enters the arena as a new cardiovascular risk factor, the translation of these findings into the clinic remains a challenging task due to the lack of evidence from large randomized clinical trials that could recommend testosterone treatment to prevent cardiovascular disease. Given the complexity of the issue, this paper aims to define all the directions of research in the field from which lessons could be learned, to connect all the lines between hypogonadism and atherosclerosis and then dwell on each to explain the evidence-so-far for that link. Finally, the paper provides a synthesis for the clinician and researcher as to what could be currently implemented in the clinic and what research remains to be done.

## 2. Connecting the Lines

**Figure 1:**
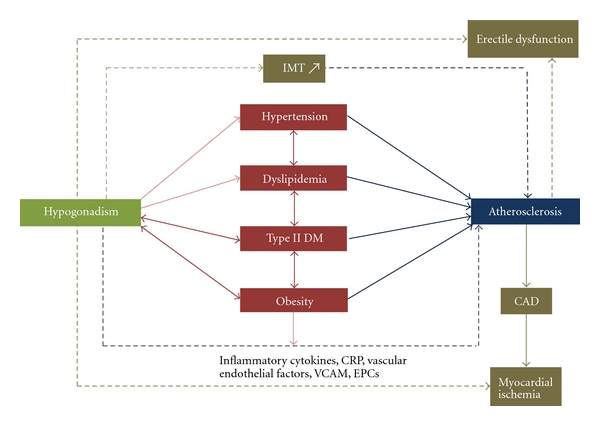
The lines connecting hypogonadism and atherosclerosis. Hypogonadism has been associated with several risk factors of atherosclerosis including obesity, Type II DM, dyslipidemia and hypertension. The relation of hypogonadism with Type II DM and obesity is most likely bidirectional. All risk factors are interrelated, and the ultimate result is increased atherosclerosis. This has been well studied in epidemiological studies, which associated low testosterone levels with increased IMT, a known marker or early atherosclerosis. Hypogonadism also contributes to the events leading to atherosclerosis by increasing inflammation and affecting endothelial function, and several other cellular mechanisms involved in the pathogenesis of atherosclerosis. In addition, low testosterone increases the susceptibility to myocardial ischemia. Erectile dysfunction is a symptom of hypogonadism, but also an end result of atherosclerosis and a predictor of CAD.

Figure 1 shows all the links between hypogonadism and atherosclerosis. Hypogonadism has been associated with several risk factors of atherosclerosis. A hypogonadal state stimulates visceral fat formation and central obesity results. Low testosterone has also been associated with the metabolic syndrome, insulin resistance, and type II diabetes mellitus [4]. An unfavorable lipid profile and hypertension have also been associated with hypogonadism [6]. The “egg and the chick” rule prevails for the relation of each of these risk factors with hypogonadism, and most likely the relation is bidirectional. On the other hand, hypogonadism has been implicated in the pathogenesis of atherosclerosis, through modulating inflammation as well as vascular endothelial function [4]. Some of these effects are modulated by visceral fat, which acts itself as an endocrine organ secreting substances that are implicated in the pathogenesis of atherosclerosis. Testosterone also has direct effects on the myocardium, and various studies have linked a hypogonadal state to myocardial susceptibility to ischemia [5]. Intima-Media Thickness (IMT) is a marker of atherosclerosis, and various studies have shown an inverse relationship between testosterone and IMT [7]. Finally, erectile dysfunction (ED) has a vascular element and an endocrine element. ED is a symptom of hypogonadism due to low testosterone. At the same time, ED can be a result of peripheral vascular disease in cases of atherosclerosis. All these links are depicted in Figure 1. We aim in this paper to simplify this complexity by discussing the evidence in each connecting line.

## 3. Research Directions

Initial observation studies in the late 19th century by Brown-Sequard on the “rejuvenating elixir” extracted from dog and guinea pig testicles [8] led to the discovery of testosterone in the 1930s [9]. Since then, testosterone has been tremendously studied in health and disease, yet the ability of this substance to prolong life remains a mystery. The relationship of testosterone to atherosclerosis is an area where this hormone can tremendously affect survival, and this is why it has been extensively studied. Current evidence on the relation of hypogonadism to atherosclerosis comes from different directions of research.

### 3.1. Epidemiological Studies

Given the known anabolic function of testosterone and its effect on increasing muscle and decreasing fat mass, numerous epidemiological and population studies correlated testosterone levels with risk factors of atherosclerosis, such as obesity, insulin resistance, and an unfavorable lipid profile. Numerous correlations were also found between testosterone levels and markers of atherosclerosis, whether imaging-related markers such as Intima-Media Thickness (IMT) or the presence of plaques and atheroma size or inflammatory markers. Furthermore, studies found an inverse relation between testosterone levels and premature coronary artery disease (CAD) among a list of other cardiovascular-related outcomes including mortality. 

### 3.2. Animal Studies

In order to investigate the pathophysiology behind the findings of epidemiological studies, basic science laboratory research is essential. The focus is to look at the pathways whereby testosterone affects the risk factors and the events leading to atherosclerosis. Within that context, most of the lessons were learned from experiments on animals. In one recent study for instance, the role of the androgen receptor (AR) on atherosclerosis was studied in mice. Atherosclerosis in AR knockouts was compared to wild type confirming the presence of both an AR-dependent and an AR-independent pathway for atheroprotection by testosterone [10]. Animal studies are also crucial in looking at the effects of exogenous testosterone administration on atherosclerosis. Testosterone can be administered in any dose, and atherosclerosis lesions can be assessed pathologically. In one recent study, dihydrotestosterone suppressed foam cell formation and attenuated atherosclerosis development in rabbits [11].

### 3.3. Basic Science Research

Apart from animal studies, basic science is carried on cell lines and using cellular and molecular techniques to decipher the actions of testosterone on the cell. In one instance, the effect of testosterone is studied on endothelial cells *in-vitro*, and in another, the expression of inflammatory markers is studied in cell lines. Such basic science research is essential to corroborate clinical observations on humans and to further understand the mechanisms leading to the observed effect.

### 3.4. Androgen Deprivation Therapy

Androgen deprivation therapy (ADT) is a commonly used treatment in prostate cancer patients based on the known role of androgens in stimulating the prostate. Prostate cancer is the most common cancer in men, and ADT was shown to be effective in improving survival and quality of life [12]. The use of ADT in prostate cancer is on the rise, and it was estimated in 2005 that half a million Americans are on ADT [13]. It is usually achieved medically using GnRH agonists or antagonists and less commonly surgically by orchiectomy. The desired serum testosterone levels after ADT are usually six times lower than the lower normal in normal young men [14]. Such medically induced hypogonadism constitutes a natural population where the effects on atherosclerosis have been studied. Many prospective studies on ADT-treated men have shown increased fat mass and different metabolic and cardiovascular perturbations [15].

### 3.5. Testosterone-Treated Patients

On the other side of the spectrum, lessons could be learned from testosterone-treated patients for a confirmed diagnosis of hypogonadism. After the clinician suspects hypogonadism, making the diagnosis is straightforward with serum testosterone levels being the single most important diagnostic test. Once a subnormal serum testosterone concentration is confirmed, treatment with exogenous testosterone is indicated. Numerous studies have been conducted on testosterone-treated patients and have documented improvement of cardiovascular risk or outcome measures in prospective studies or better outcomes than age-matched controls in cross-sectional studies. These studies also look into the different types, doses, and delivery modes of testosterone in terms of improvement of cardiovascular-related outcomes and frequency of adverse effects.

### 3.6. Randomized Clinical Trials

Randomized clinical trials (RCTs) are the needed tool to allow recommending testosterone treatment to prevent or treat atherosclerosis. This goal is still far from reach, with the current literature being poor in large well-designed RCTs that can provide solid evidence. A systematic review and meta-analysis was performed in 2007, and it reported only 30 RCTs, which mostly had small numbers, poor methodology, and inconsistent results [16]. More recently, two RCTs from Italy and Moscow with 50 and 184 subjects, respectively, showed improved indicators of the MetS and decrease in some inflammatory markers with testosterone treatment as compared to placebo [17, 18].

## 4. Hypogonadism and Atherosclerotic Risk Factors

We already established that hypogonadism has been associated with several risk factors of atherosclerosis. The relationship between testosterone levels and the risk factor is frequently bidirectional, and the different risk factors are often interrelated. We choose in this paper to discuss four main risk factors whose relations with hypogonadism have been well studied.

### 4.1. Type II Diabetes Mellitus, Insulin Resistance, and the Metabolic Syndrome

Type II Diabetes Mellitus (DM II) is one of the most serious risk factors of atherosclerosis. Several epidemiological studies confirmed that low testosterone is associated with DM II and the Metabolic Syndrome (MetS). Even after age-adjustment, cross-sectional studies showed significantly lower testosterone levels in DM II patients compared to normal controls [19, 20]. Other studies also showed similar associations between low testosterone and the MetS [21, 22]. In prospective studies, low testosterone predicted future development of DM II and the MetS [23]. In most of these studies, total testosterone is more associated with DM II and the MetS than free testosterone. This is mostly because the relation is mediated by low levels of Sex Hormone-Binding Globulin (SHBG), which lead to a low total testosterone. Low SHBG has been independently associated with insulin resistance [24], and it has been suggested that SHBG might have a direct causing role of insulin resistance [25]. Another evidence comes from prostate cancer patients who are on ADT. These patients have a higher prevalence of insulin resistance and hyperglycemia as well as higher incidence of DM II [26]. On the other hand, testosterone treatment of DM II patients resulted in favorable outcomes such as improved glycemic control [27] and decreased need for insulin in insulin-dependent patients.[28] There is also a dose-effect relationship between the dose of testosterone used and degree of insulin resistance [29]. The TIMES2 study recently showed that six-month treatment with transdermal testosterone of hypogonadal men with DM II and/or MetS resulted in improved glycemic control, body composition, cholesterol levels, lipoprotein a, and erectile dysfunction as compared to the control group [30]. More importantly, there was no significant difference in the frequency of adverse events between the two groups and the majority of the adverse events were mild or moderate [30]. It has been well established that the favorable outcomes of higher testosterone in DM II patients are due to improved insulin resistance, but there has also been little evidence from animal studies on a direct effect of testosterone on the pancreas [31]. This is contradicted, however, by the lack of association between hypogonadism and Type I Diabetes Mellitus (DM I) suggesting that the effect of low testosterone is not on hyperglycemia and the pancreas *per se*. A large part of the association between hypogonadism and the MetS is due to the effect of low testosterone on adipocyte biology and subsequent obesity, which contributes to all the features of the MetS such as insulin resistance, dyslipidemia, and hypertension.

### 4.2. Obesity and Increased Adiposity

Obesity is strictly involved in the pathophysiology of the relationship between hypogonadism and DMII or MetS. Furthermore, the relationship of obesity and insulin resistance with hypogonadism is most probably bidirectional. The best evidence of increased adiposity in hypogonadism comes from ADT patients who accumulate body fat within three months of starting treatment [32]. Incident DM II is also higher in ADT patients [33]. Additional evidence comes from testosterone-treated patients in whom reduction of fat mass has been documented [34]. The increased visceral fat that accumulates in hypogonadism serves as an endocrine organ that releases inflammatory cytokines that contribute to insulin resistance, DM II and the MetS [35]. It has also been suggested that androgens not only attenuate adipogenesis, but also inflammation *per se* [36]. This is very important in terms of vascular health and the direct effects of testosterone on the events leading to atherosclerosis as will be discussed in a subsequent section of this paper. The reverse relation of obesity and hypogonadism also holds true. Testosterone levels correlate inversely with weight changes [37, 38] and the MetS predicts future incidence of hypogonadism [39]. This relation is in its turn potentially mediated by increased release of inflammatory cytokines, which inhibit the hypothalamo-pituitary-gonadal axis at different levels [40]. Adipose tissue also contains aromatase, which converts testosterone to 17*β*-estradiol levels. Estradiol seems to inhibit luteinizing hormone secretion since treatment of obese men with aromatase inhibitors increases testosterone levels [41]. In summary, obesity can cause hypogonadism, and hypogonadism can cause obesity, and in both cases a state of increased visceral fat is a major risk factor for atherosclerosis, whether through a direct effect on the vasculature or through increasing the incidence and magnitude of other risk factors of atherosclerosis such as insulin resistance, dyslipidemia, and hypertension. For further reading on the link between obesity and hypogonadism, we suggest consulting the recent reviews by Saad and Gooren and Traish et al. [42, 43].

### 4.3. Dyslipidemia

**Figure 2:**
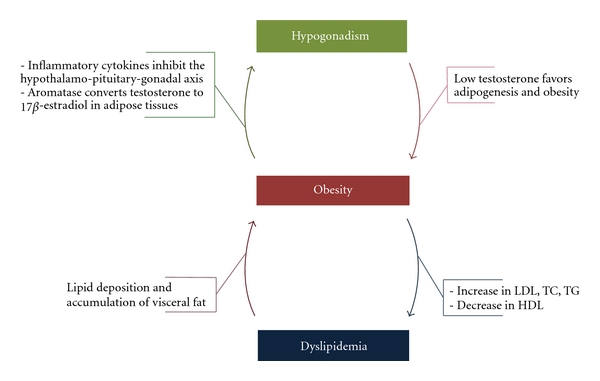
The vicious cycle of hypogonadism, obesity, and dyslipidemia. Low testosterone can cause obesity and fat accumulation, which results in dyslipidemia and further deposition of visceral fat. Obesity can also decrease testosterone levels through conversion of aromatase to estradiol in the adipose tissue and through the release of inflammatory cytokines that can inhibit the hpothalamo-pituitary-gonadal axis at multiple levels.

Lipid delivery to the vessel wall is a crucial component of the pathophysiology of atherosclerosis. This is why an unfavorable lipid profile is another major independent risk factor of atherosclerosis. Epidemiological studies, studies on testosterone-treated patients, and studies on ADT patients showed that low testosterone results in an unfavorable lipid profile. Such a profile is usually an increase in triglycerides, total cholesterol, LDL, and oxidized LDL and a decrease in HDL [6]. All these result in endothelial dysfunction, oxidative stress, and inflammation, all of which are mechanistic processes in atherosclerosis. It has also been shown that an unfavorable lipid profile can contribute to the development of the MetS and DM II [40, 44]. Cross-sectional studies have confirmed that total and LDL cholesterol inversely correlate with testosterone levels [45–47]. Testosterone treatment in numerous studies also resulted in a decrease in total and LDL cholesterol [48–50]. On the other hand, HDL positively correlates with testosterone levels in cross-sectional studies [51], yet evidence from the effects of testosterone treatment on HDL remains controversial [52–54]. Lipoprotein a is an established risk factor of atherosclerosis [55]. In a recent study on ageing hypercholesterolemic men, clinically significant elevations of lipoprotein a were found to be more prevalent in men with low testosterone [56]. We had also mentioned that the testosterone treatments of DMII/MetS patients in the TIMES2 study resulted in lower lipoprotein a [30]. Mechanistic studies have suggested that the change in lipid profile is related to increased adiposity in hypogonadal men, with subsequent increase in estradiol levels due to aromatization. Estradiol decreases testosterone levels and at the same time can result in the unfavorable lipid profile discussed [57]. Increased HDL can be explained in this context by the increased estradiol levels, given the reports of higher HDL in women compared to men [58]. There seems to be a vicious cycle involving hypogonadism, obesity and the unfavorable metabolic profile. This profile of dyslipidemia, insulin resistance, and subsequent DM II perpetuates the metabolic syndrome through increased adiposity, which results in further decrease in testosterone levels (Figure 2).

### 4.4. Hypertension

Hypertension is an important risk factor for atherosclerosis due to its involvement in the pathophysiology of vascular dysfunction through endothelial cell injury. Hypertension is yet another risk factor that has been associated with low testosterone levels [59]. This association might sound counter-intuitive given evidence that anabolic androgens used mostly in young men for body-building purposes increase the risk of hypertension [60]. However, studies have suggested that replenishment of testosterone to normal levels in hypogonadal men results in decreased blood pressure [53, 61]. Studies to identify an androgen-related-independent pathway for these improvements in blood pressure are lacking, and it is very probable that the observed improvements in blood pressure in these testosterone-treated men are due to the decreased adiposity and improved metabolic profile in general. However, several possible mechanisms have been suggested to explain the antihypertensive effects of testosterone. Kumanov established that plasma endothelin 1 (ET1) levels are elevated in males with hypogonadism, and testosterone treatment decreases ET1 [62, 63]. Another hypothesis is that testosterone treatment of hypogonadism normalizes the contractile RhoA/Rho-kinase (ROCK) signaling pathway, which is upregulated in hypogonadal states [64]. Other mechanisms involve decreased nitric oxide synthase (NOS) and increased asymmetric dimethylarginine (ADMA) in a hypogonadal state, which are reversed through testosterone administration [65–67].

## 5. Hypogonadism and Atherosclerotic Events

Atherosclerosis is a complicated process with endothelial dysfunction, oxidative stress, lipid deposition, and inflammation involved. Direct effects of hypogonadism on the pathophysiology of atherosclerosis have been studied in animals and *in-vitro*. This has contributed tremendously to our understanding of different mechanisms through which low testosterone can accelerate the atherosclerotic process. Initially, it was hard to explain why testosterone levels inversely correlated with atherosclerosis in men and women in large epidemiological studies such as the Rotterdam study [68]. This sounded counter-intuitive since females are known to be protected from atherosclerosis by estrogens. Androgens on the other hand have opposite effects, which favor the formation of atherosclerosis. For instance, testosterone increased the uptake of cholesterol and formation of the foam cell of the fatty streak through androgen receptors, which are expressed in the macrophages [69]. Additionally, testosterone increased the apoptosis of endothelial cells in men, which also favors atherosclerosis [70]. Given the results of large epidemiological studies including the Rotterdam study, all showing that low levels of testosterone accelerate atherosclerosis, further animal and *in-vitro* studies were looking into the mechanisms of that benefit of testosterone to vascular health. Castrated rabbits that were fed with cholesterol rich diet had less atherosclerosis when treated with testosterone [71]. Testosterone supplementation also had an inhibitory effect on neointima formation and plaque development [72, 73]. The decreased lipid deposition on testosterone supplementation remained in testicular feminized mice that do not have neither testosterone nor an androgen receptor [74]. This suggested that testosterone acts through an androgen-receptor-independent pathway. Similar studies yielded more and more pathways and mechanisms that could explain the reason behind the association of higher testosterone with less atherosclerosis. A summary of these mechanisms is elucidated below.

### 5.1. Inflammation

Inflammation is an essential component of atherosclerosis. Inflammatory cytokines have a role in the early development of the atherosclerotic plaque. Various small studies suggest a role for testosterone in immune modulation and in decreasing the inflammatory component in atherosclerosis [18, 75]. Testosterone replacement in men has been shown to decrease the levels of endogenous inflammatory cytokines implicated in atherosclerosis such as TNF-*α*, IL-6, and IL-*β* [76]. It also increased atheroprotective cytokines such as IL-10 *in-vitro* as well as in treated hypogonadal men [76, 77]. C-reactive protein (CRP) is a marker of general inflammation and is produced by the liver in response to IL-6. Levels of CRP are used as a marker of atherosclerotic cardiovascular disease. There seems to be an inverse correlation between CRP and testosterone levels [78, 79]. Testosterone replacement has not been shown to affect CRP levels so far [79, 80]; however, ADT patients showed an increased CRP in one study [26]. While *in-vitro* studies on cell lines serve a great role in identifying the role of testosterone on expression of inflammatory markers, it is hard to make rigid conclusions from *in-vivo* studies on men. This is because the relationship between testosterone levels and inflammation can be confounded by obesity, the MetS, and DM II, all of which can be caused by hypogonadism and at the same time can contribute to an increased state of inflammation. Therefore, conclusive evidence could only come by complementing observations on men that are duplicated and observed in large population groups with *in-vitro* experiments that could explain the observations in men and can be duplicated by several basic scientists. This goal is still unreached so far.

### 5.2. Atherosclerosis Mediating Molecules

Vascular Cell Adhesion Molecule (VCAM) is one of the molecules that permits the migration of the macrophage into the vascular wall and is hence a major contributor to the formation of the fatty streak, the initial lesion in atherosclerosis. A study on male CAD patients showed that VCAM levels inversely correlated with testosterone levels [81]. However, testosterone replacement in hypogonadal men did not affect serum levels of VCAM [80]. *In-vitro* studies on human aortic endothelial cells showed that testosterone inhibits TNF-*α*-induced VCAM expression [82]. Further research will need to study VCAM levels in testosterone replacement patients in larger doses and over longer periods of treatment.

### 5.3. Endothelial Function and Vascular Health

Endothelial function is one important element of vascular health. Any injury to the endothelium or perturbation in its function can result in change of vascular tone, hypertension, formation of a thrombus, or initiation of a new atherosclerotic plaque. Endothelial dysfunction can be assessed clinically with surrogate markers such as flow-mediated dilatation in the brachial artery prior to the development of atherosclerosis. Cross-sectional studies associated a low testosterone level in men with endothelial dysfunction [83]. Prospective studies on testosterone-treated men with CAD showed that testosterone was able to improve their endothelial function [84]. Yet, the effect of testosterone replacement of hypogonadal men on endothelial function has yielded controversial results, which varied by the dosage form of testosterone [85, 86]. Other studies assessed the effect of testosterone levels on arterial stiffness, which is an independent predictor of CAD [87]. Epidemiological studies inversely correlated testosterone levels with arterial stiffness [88], and testosterone replacement decreased arterial stiffness [85]. Endothelin-1, a vasoconstrictive hormone implicated in hypertension and CAD, was also studied. Its levels were elevated in hypogonadal men, and it was reduced with testosterone treatment [89]. Endothelin-1 is produced in aortic endothelial cells, and it is thought that testosterone upregulates the expression of the hormone in these cells [89]. Endothelial Progenitor Cells (EPCs) have also been studied in CAD, and circulating EPCs were shown to have a role in maintaining the integrity of the endothelium and were associated with carotid IMT [90]. Hypogonadal men had low levels of EPCs that could be increased by exogenous testosterone supplementation [91]. One study looked at the expression and function of the AR in human EPCs *in-vitro* as well as the effect of testosterone on their function [92]. Androgens stimulated human EPC proliferation and colony formation in concentrations similar to those present in humans through an AR-dependent pathway [92].

### 5.4. Myocardial Health

Although atherosclerosis is a diffuse disease, the biggest fear is its effect on the heart. In addition to the risk factors and events involved in CAD that we discussed, hypogonadism also negatively affects myocardial health and can potentially worsen myocardial ischemia in the setting of CAD. The antianginal properties of testosterone were first reported in the 1940s [93–95]. Depression of the ST segment on the electrocardiogram is a marker of myocardial ischemia. Numerous studies have reported decreased anginal episodes and reduced ST depression on treatment with testosterone [5, 96, 97]. Although part of these observations is explained by the effect of testosterone on vascular tone discussed above, it has also been suggested that testosterone has direct effects on ventricular repolarization of the myocardium [98]. There is accumulating evidence that testosterone protects from myocardial ischemia. Recent small randomized trials showed improved exercise capacity and increased time to ST depression as well as decreased frequency of anginal attacks [5, 99]. Another RCT also showed that long-term testosterone treatment can improve exercise capacity and glucose metabolism in elderly men with stable congestive heart failure (CHF) [100]; hence, testosterone replacement is being studied as a treatment for heart failure, given its anabolic effects on muscle mass and strength [101].

## 6. Hypogonadism and Atherosclerotic Surrogate End Points

We discussed so far the lines connecting hypogonadism to each of the risk factors of atherosclerosis and the events involved in its pathophysiology. Clinical trials and other epidemiological studies correlate testosterone levels to different surrogate end points for atherosclerosis. Surrogate end points are crucial to accurately assess outcome in randomized placebo-controlled trials. Different end points have been used with differential levels of evidence for each.

### 6.1. Carotid Intima Media Thickness (IMT)

Carotid IMT is one of the most commonly used surrogate end points of atherosclerosis. It is used as a subclinical marker of atherosclerosis, and it is a common outcome measure in most studies on disease progression and effects of treatment including testosterone supplementation. For instance, low testosterone levels were related to carotid IMT independent of other cardiovascular risk factors [7], and testosterone replacement therapy reduced carotid IMT independently from BMI [102].

### 6.2. Flow Mediated Dilation (FMD)

FMD is a measure of vascular tone and is usually performed in the brachial artery. An increased FMD indicates endothelial dysfunction, which has been shown to predict CAD [103]. We have established earlier that endothelial dysfunction and arterial stiffness have been associated with hypogonadism. FMD is one surrogate measure used to assess the endothelial disruption caused by a low testosterone.

### 6.3. Erectile Dysfunction (ED)

 Erectile dysfunction is one of the symptoms of hypogonadal hypogonadism accompanying the decreased libido caused by decreased serum testosterone. However, ED is also an early marker of generalized atherosclerosis and a predictor of cardiovascular events [104]. ED cannot be used as a surrogate end point for atherosclerosis in studies looking at testosterone, because the effect itself is confounded by the low testosterone levels. Nevertheless, it is important to note that the pathophysiology of ED in hypogonadal men could be due to the direct effect of a low testosterone and also to the atherosclerosis caused by hypogonadism.

### 6.4. Clinical Events

Clinical events related to morbidity and mortality from atherosclerosis are also used as end point measures in studies on hypogonadism and atherosclerosis. These include cardiovascular events, cerebrovascular events, and mortality. For the majority of large longitudinal cohort studies such as the Framingham study, the Caerphilly study, and the Tromso study, testosterone levels were not associated with incident cardiovascular disease [105]. Whether testosterone levels are associated with the incident strokes and transient ischemic attacks has been controversial [105]. When mortality is taken as an outcome measure in longitudinal studies, the findings have been more consistent. Lower testosterone levels predict all cause mortality as well as mortality from CVD [105].

## 7. Safety of Exogenous Testosterone

If testosterone is to be considered for treatment and prevention of atherosclerosis and cardiovascular disease, it has to be safe. A lot is known about the side effects from the abuse of androgens in athletes, the most important of which is sudden death [14]. Other side effects include mainly cardiac events such as arrhythmias, cardiomypopathy, and myocardial infarctions, as well as noncardiac adverse effects such as polycythemia, acne, and hepatotoxicity [14]. Nevertheless, athletes use doses of androgens that are several times higher than the doses used medically in testosterone replacement therapy [14]. Hence, such supraphysiological levels of testosterone can cause side effects that are not caused by replacement of testosterone to physiological levels or slightly higher. Different metanalyses and systematic reviews have looked at the safety of testosterone treatment when used in the usual pharmacological doses. Possible adverse effects include increase in prostate events, increase in hemoglobin and hematocrit, and a small decrease in HDL cholesterol, but the evidence is still poor and with unknown clinical significance due to short followup [106–108].

## 8. Summary

**Figure 3:**
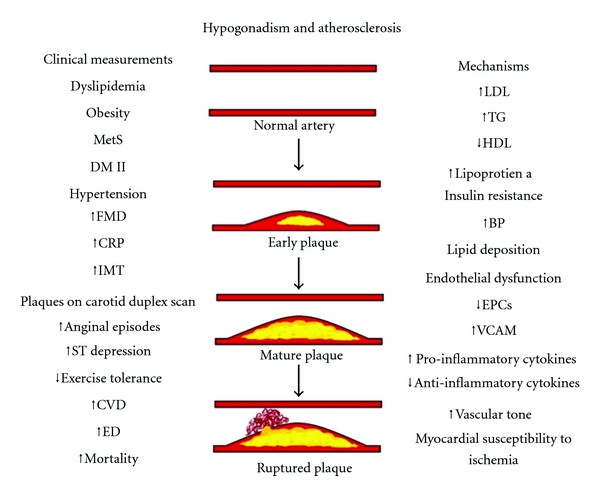
Clinical indicators and mechanistic processes involved in the relation of hypogonadism and atherosclerosis at each level of the pathogenesis of the disease.

Hypogonadal hypogonadism is a common disease especially in elderly men who are at high risk of atherosclerotic cardiovascular disease. Low testosterone is a risk factor for atherosclerosis. Not only does it cause several of its risk factors, but it also accelerates the events involved in the pathophysiology of atherosclerosis. Studies have confirmed the link between testosterone levels and several surrogate end points of atherosclerosis. Different clinical indicators of atherosclerosis have been associated with hypogonadism, and different mechanistic processes involved in atherosclerosis have been linked to hypogonadism.  Figure 3 shows the progression of atherosclerosis and summarizes the clinical indicators studied and mechanistic processes involved at each level of the disease as discussed in this paper.

## 9. Recommendations for the Researcher and Clinician

Over the past two decades, the interest in androgens has stimulated a lot of research that increased our understanding of their role in atherosclerosis. Evidence comes from different directions of research, yet gaps are still large in different areas, and more research is to be done. Particularly, there is a need for large randomized placebo-controlled clinical trials to assess the efficiency and safety of testosterone treatment in healthy men to prevent or delay atherosclerosis. Also more basic science research is essential to understand the mechanisms behind the involvement of testosterone in the risk factors and events leading to atherosclerosis.

At the moment, testosterone replacement is only recommended for patients with a confirmed diagnosis of hypogonadal hypogonadism. Clinicians are highly recommended to recognize the clinical features of hypogonadism and to screen for it and treat it to improve quality of life and prevent atherosclerosis. Current research is not enough to recommend testosterone supplementation for patients who do not have hypogonadism, in the absence of large randomized placebo-controlled trials.
